# Traditional Chinese Medicine Fufang-Zhenzhu-Tiaozhi capsule prevents renal injury in diabetic minipigs with coronary heart disease

**DOI:** 10.1186/s13020-022-00648-x

**Published:** 2022-08-30

**Authors:** Lixia Song, Ke Wang, Jianying Yin, Yiqi Yang, Bo Li, Dongxing Zhang, Hong Wang, Weixuan Wang, Wenjing Zhan, Caijuan Guo, Zhanhui Gu, Lexun Wang, Zhihuan Zeng, Weijian Bei, Xianglu Rong, Jiao Guo

**Affiliations:** 1 Guangdong Metabolic Disease Research Center of Integrated Chinese and Western Medicine, Guangdong, China; 2 Key Laboratory of Glucolipid Metabolic Disorder, Ministry of Education of China, Guangdong, China; 3 Key Unit of Modulating Liver to Treat Hyperlipemia SATCM (State Administration of Traditional Chinese Medicine), Guangdong, China; 4 Institute of Chinese Medicinal Sciences, Guangdong TCM Key Laboratory Against Metabolic Diseases, Guangdong Pharmaceutical University, Guangdong, China; 5 Department of Cardiovascular Diseases, the First Affiliated Hospital of Guangdong Pharmaceutical University, Guangdong, China

**Keywords:** Fufang-Zhenzhu-Tiaozhi (FTZ), Diabetes, Renal injury, Oxidative stress, Apoptosis

## Abstract

**Background:**

Renal injury is one of the common microvascular complications of diabetes, known as diabetic kidney disease (DKD) seriously threatening human health. Previous research has reported that the Chinese Medicine Fufang-Zhenzhu-Tiaozhi (FTZ) capsule protected myocardia from injury in diabetic minipigs with coronary heart disease (DM-CHD). And we found significant renal injury in the minipigs. Therefore, we further investigated whether FTZ prevents renal injury of DM-CHD minipig and H_2_O_2_-induced oxidative injury of HK-2 cells.

**Methods:**

DM-CHD model was established by streptozotocin injection, high fat/high-sucrose/high-cholesterol diet combined with balloon injury in the coronary artery. Blood lipid profile, fasting blood glucose (FBG), and SOD were measured with kits. The levels of blood urea nitrogen (BUN), serum creatinine (Scr), urine trace albumin (UALB), urine creatinine (UCR) (calculate UACR), cystatin (Cys-C), and β-microglobulin (β-MG) were measured by ELISA kits to evaluate renal function. TUNEL assay was performed to observe the apoptosis. qPCR was used to detect the mRNA expression levels of HO-1, NQO1, and SOD in kidney tissue. The protein expressions of Nrf2, HO-1, NQO1, Bax, Bcl-2, and Caspase 3 in the kidney tissue and HK-2 cells were detected by western blot. Meanwhile, HK-2 cells were induced by H_2_O_2_ to establish an oxidative stress injury model to verify the protective effect and mechanisms of FTZ.

**Results:**

In DM-CHD minipigs, blood lipid profile and FBG were elevated significantly, and the renal function was decreased with the increase of BUN, Scr, UACR, Cys-c, and β-MG. A large number of inflammatory and apoptotic cells in the kidney were observed accompanied with lower levels of SOD, Bcl-2, Nrf2, HO-1, and NQO1, but high levels of Bax and Cleaved-caspase 3. FTZ alleviated glucose-lipid metabolic disorders and the pathological morphology of the kidney. The renal function was improved and the apoptotic cells were reduced by FTZ administration. FTZ could also enhance the levels of SOD, Nrf2, HO-1, and NQO1 proteins to promote antioxidant effect, down-regulate the expression of Bax and Caspase3, as well as up-regulate the expression of Bcl-2 to inhibit cell apoptosis in the kidney tissue and HK-2 cells.

**Conclusions:**

We concluded that FTZ prevents renal injury of DM-CHD through activating anti-oxidative capacity to reduce apoptosis and inhibiting inflammation, which may be a new candidate for DKD treatment.

**Graphical Abstract:**

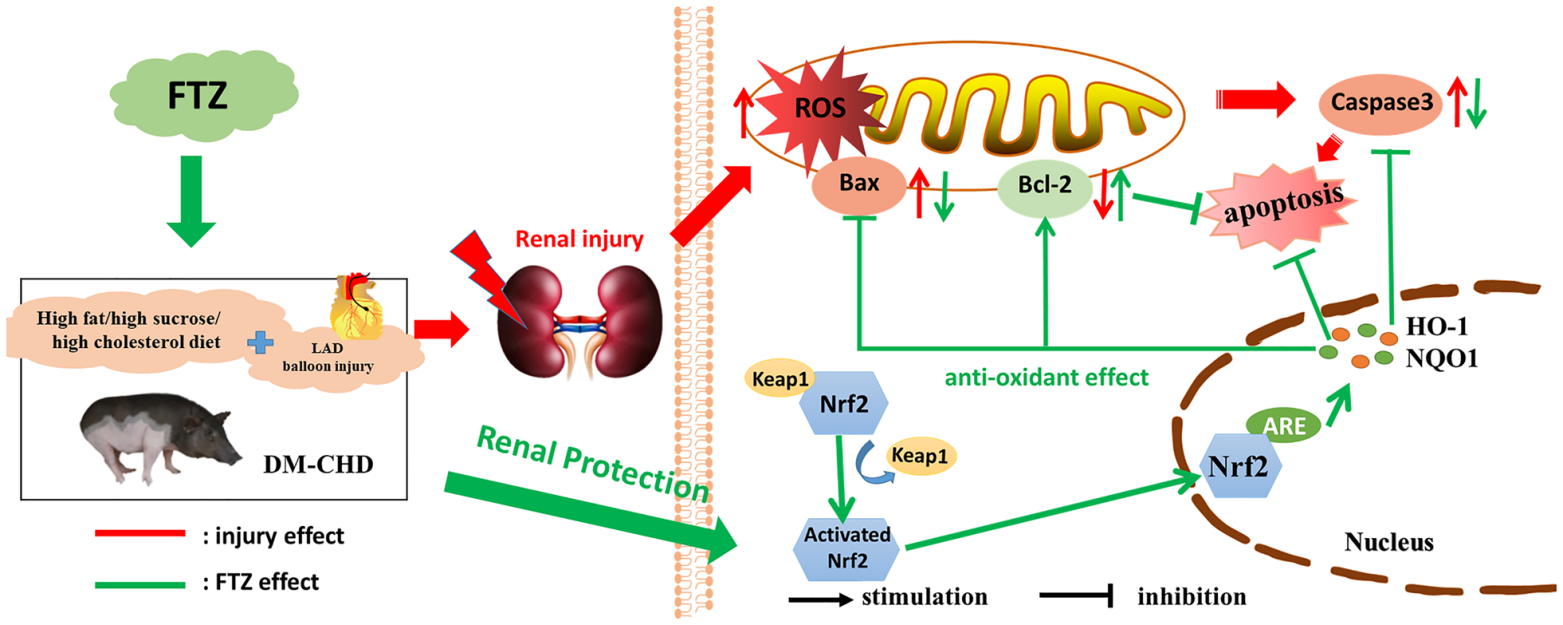

## Introduction

In recent years, with the improvement of living standards, obesity, genetic susceptibility, urbanization, and aging, the incidence of clinical diabetic kidney disease (DKD) has been rising rapidly [1]. According to statistics, there were 463 million people with diabetes mellitus (DM) worldwide in 2019. By 2045, this number will increase to 700 million [2].

The toxicity of diabetic hyperglycemia affects all organs of the body, among which the vascular system is the most affected, and it can cause a variety of microvascular complications [3–5]. Diabetes-induced renal injury is a common and serious microvascular complication in DKD, which has become the most important cause of the end-stage renal disease (ESRD), its severity is second only to cardiovascular disease [6, 7]. Although many therapeutic drugs have been developed for the treatment of DKD, such as RAAS inhibitor, SGLT2 inhibitor, antioxidant, Nrf2 activator, and NOX inhibitor, these drugs still have many deficiencies, such as increased susceptibility to genital and urinary tract infections, increased risk of heart failure, and effects are not specific [8–15]. The DKD has not been alleviated, which has increased the difficulty and economic burden of diabetes treatment [16].

Reactive oxygen species (ROS) is an important driver of diabetic complications, and the central mechanism mediating these diabetic complications initially focuses on the dysregulation of ROS produced by mitochondria [17, 18]. Nrf2, as a key regulator of antioxidant and cell protective agent, is mainly activated in response to oxidative stress [19, 20]. Nrf2 binds to Kelch-like epichlorohydrin-associated protein 1 (KEAP-1) in the cytoplasm under physiological conditions. When the body continues to be exposed to high glucose, ROS is overproduced in the body, Nrf2 no longer binds to KEAP-1, and Nrf2 enters the nucleus and binds to antioxidant reaction elements to complete transcription and up-regulate hemoglobin oxygenase-1 (hypocretin-1) (HO-1) and Quinone oxidoreductase (NAD(P)H Quinone Dehydrogenase 1) (NQO1), thereby improving cellular oxidative stress. At the same time, excessive ROS stimulates lipid oxidation of mitochondria, destroying the structure and function of mitochondria. Apoptosis-inducing factors are released from the cytoplasm to stimulate the transport of pro-apoptotic proteins such as Bax and Caspase 3, thus leading to apoptosis [21, 22].

As a signal, ROS regulates the responses of multiple intracellular pathways, causing a series of reactions such as severe fibrosis, excessive oxidative stress, and severe apoptosis of kidney tissue [23]. Thus, the focus now has been on specific mechanism-based strategies that can target oxidative stress pathways to improve the outcome of disease burden. SOD is one of the key antioxidant enzymes which could defend against ROS-induced oxidative stress in vivo [24]. Nrf2 is a redox-sensitive transcription factor, developing an important function in oxidative stress and tissue injury [20].

Metformin is an antidiabetic drug prescribed to treat type II diabetes [25]. Atorvastatin is an oral drug that is effective in lowering triglycerides, and potent in reducing LDL-C. Metformin combined with atorvastatin is an effective treatment for diabetic cardiomyopathy. However, it is limited in the treatment of DKD with its side effects [26, 27].

Fufang-Zhenzhu-Tiaozhi (FTZ), a patented Chinese herbal medicine prescription, is composed of eight traditional Chinese medicinal (TCM) herbs, including *Rhizoma coptidis, Radix Salvia Miltiorrhiza, Radix Notoginseng, Fructus Ligustri Lucidi, Herba Cirsii Jeponici, Cortex Eucommiae, Fructus Citri Sarcodactylis, and Radix Atractylodes Macrocephala* [28, 29]. Previous studies demonstrated that FTZ has the effects of lowering blood lipid, anti-inflammatory, anti-oxidative stress, and improving insulin resistance, and has a good prevention and treatment effect on glycolipid metabolism disease [30–34]. FTZ could also protect myocardia from injury in diabetes mellitus with coronary heart disease (DM-CHD) in the minipigs model [35], and we also found that there was renal injury in the DM-CHD model. Therefore, we performed this study to explore whether FTZ has a protective effect on the complications of renal injury caused by the DM-CHD through the antioxidative signal pathway.

## Methods

### Materials and reagents

Test kits of glucose (GLU), triacylglycerol (TG), total cholesterol (TC), high-density lipoprotein cholesterol (HDL-C), and low-density lipoprotein cholesterol (LDL-C) were purchased from Shanghai Rongsheng Biotech Co. Ltd. (Shanghai, China). Hematoxylin (HHS32) solution and eosin Y solution (HT110332) were purchased from Sigma-Aldrich (St. Louis, MO, USA). A periodic acid-Schiff (PAS) staining kit was purchased from Leagene Biotechnology (Beijing, China). Kits for blood urea nitrogen (BUN), serum creatinine (Scr), urine trace albumin (UALB), urine creatinine (Ucr) (calculate UACR), superoxide dismutase (SOD) kit, lactic dehydrogenase (LDH) kit, and Albumin kit were supplied by Nanjing Jiancheng Bioengineering Institute (Nanjing, China). Cystatin (Cys-c) Elisa kit and β-microglobulin (β-MG) Elisa kit were measured by using ELISA kits according to the manufacturer's instructions (Jiangsu Feiya Biological Technology Co. Ltd., China). One-step TUNEL cell apoptosis detection kit, RIPA buffer, ROS kit, and BCA Protein Assay Kit were purchased from Beyotime Biotechnology Co. Ltd. (Beijing, China). RNAiso Plus, Prime Script RT reagent kit with gDNA Eraser, and TB Green Premix Ex Taq II were purchased from Biomedical Technology Co., Ltd (Japan).

Antibodies against Nrf2 (ab89443), HO-1 (ab13248), NQO1 (ab2346), Bax (ab104156), Caspase3 (ab13847), Cleaved-caspase3 (ab49822) and GAPDH (ab8245) were purchased from abcam (Cambridge, UK). Antibody against β-actin (#4970 s) was purchased from Cell Signaling Technology (Beverly, MA, USA). Antibody against Bcl-2 (Bs20351r) was obtained from Bioss Biotechnology Co., Ltd. (Beijing, China).

### Animals

**Fig. 1:**
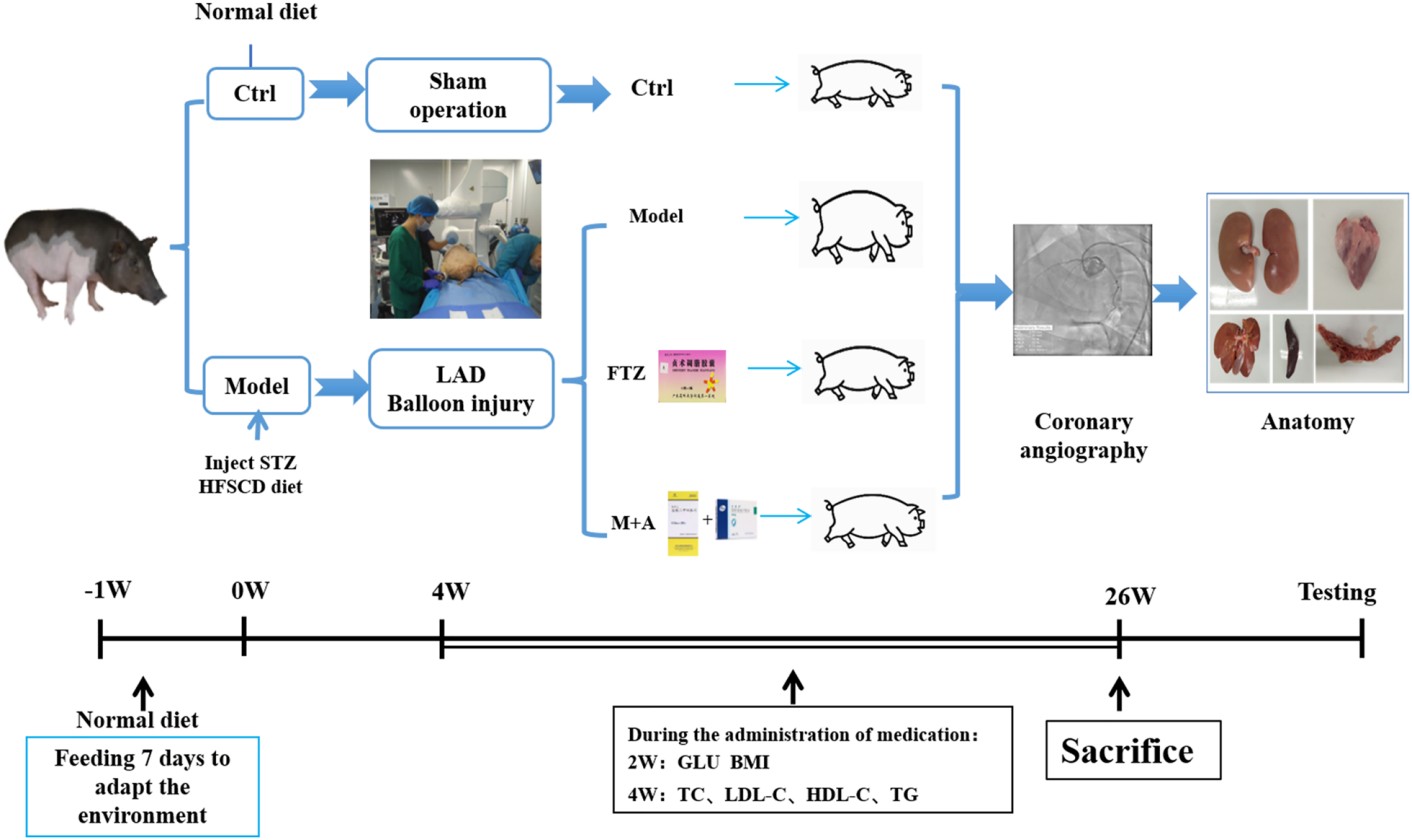
Animal experiment process

All animal studies were conducted following the ARRIVE (Animal Research: Reporting of in Vivo Experiments) guidelines for reporting experiments involving animals. The animals were kept under monitored standard laboratory conditions, complying with the European Union guidelines (directive 2010/63/EU), and the procedures were approved by the Research Ethical Committee of Guangdong Pharmaceutical University (Guangzhou, China). Eighteen castrated male Chinese Wuzhishan minipigs were supplied by Guangdong Laboratory Animals Monitoring Institute, Guangzhou, China (Certification: SYXK Guangdong 2018–0125). Pigs weighing 9–12 kg and 11–12 weeks old were used in this study. They were housed in single pens under controlled conditions (temperature between 24 °C and 26 °C, relative air humidity 30–70%) and fed twice a day with a total daily amount of 3% of bodyweight. Filtered tap water was available randomly. Animal treatment is shown in Fig. 1.

### Animal groups and treatment

After 1 week of adaptive feeding, eighteen minipigs were randomly divided into the control group (n = 4) and the DM group (n = 14). The DM group was developed by feeding an HFSCD and induced by STZ, while the control group received a normal diet (ND) and injected with citric acid-sodium citrate buffer. After feeding for 4 weeks, the DM group was subjected to left coronary artery interventional balloon injury surgery. After surgery, the DM group was divided into model group, FTZ group, and M + A (Metformin + Atorvastatin) group. At the following 4 to 26 weeks, the control group continued to feed with ND, while the model group, FTZ group, and M + A group continued to feed with HFSCD. The FTZ group was given 1.2 g/kg body weight of FTZ extracts (It was equal to 2 times of the clinic human dose.), administered with meals once a day. Metformin (57.08 mg/kg/d, according to the daily dose of Metformin in the clinical treatment of 2.550 g/60 kg) and Atorvastatin (0.92 mg/kg/d, according to the daily dose in the clinical treatment of 40 mg/60 kg) were given to the M + A group minipigs. During this period, blood samples were collected from the auricular veins using heparinized tubes monthly. At the end of the experiment, the kidney tissues of pigs were collected, frozen on dry ice, and then stored at − 80 °C.

### Biochemical marker determination

The plasma samples were isolated by centrifugation at 3500 rpm for 15 min at 4 °C, and the separated plasma was frozen at −80 °C for further analysis. Kits were used to determine plasma levels of GLU, TG, TC, HDL-C, and LDL-C. Biomarkers of renal function, such as BUN, Scr, Ucr, UALB, Cys-c, and β-MG were measured by the manufacturer's instructions. Furthermore, the Urinary Albumin Creatinine Ratio (UACR, UACR = UALB/Ucr) was calculated.

### Histological analysis

The kidney tissue was fixed in 4% paraformaldehyde for 36 h, dehydrated with graded alcohol, embedded in paraffin, and then sliced into 4 μm-thick sections. These sections were stained with H&E staining and PAS staining to assess histopathological changes and glycogen deposition. Sections were mounted and observed under a microscope (PerkinElmer, Vectra 3, USA).

### qPCR

The kidney tissues were preserved at − 80 °C before RNA extraction. Total RNA was extracted by using the RNAiso Plus following the manufacturer’s protocol (9108, TaKaRa, Japan). cDNA synthesis was carried out with Prime Script TMRT reagent Kit with gDNA Eraser (RR047A, TaKaRa, Japan). cDNA subsequently underwent quantitative real-time polymerase chain reaction (PCR) using the sequences of the following primers:

HO-1: Forward primer (5′ → 3′): GGCATCCGACATCCGCAAGAG,

Reverse primer (5′ → 3′): CACCTGGGAGAGGACGCTGAG.

NQO1: Forward primer (5′ → 3′): GTGGAAGCCGCAGACCTTGTG,

Reverse primer (5′ → 3′): GCACACGTTCAAACCAGCCTTTC.

SOD: Forward primer (5′ → 3′): GAAGATTCTGTGATCGCCCTCTCG,

Reverse primer (5′ → 3′): TTCATTTCCACCTCTGCCCAAGTC.

β-actin: Forward primer (5′ → 3′): GCGACTGCGCCCCATAAAAC,

Reverse primer (5′ → 3′): ATGGCGAACTGGTAGCGGTG.

### HK-2 cell-based assay

The human proximal tubular cell line HK-2 was purchased from the American Type Culture Collection (Manassas, VA, USA) and cultured in the DMEM F-12 with 10% fetal bovine serum (Gibco), 1% penicillin (Gibco), and 1% streptomycin (Gibco), under standard conditions (37˚C, 5% CO_2_). Cell viability was measured using Cell Counting Kit-8. Briefly, trypsin was used to digest the HK-2 cells after being grown to 90%. Moreover, the cells were seeded in the 96-well culture plate with a density of 5 × 10^4^ cells per/well. After 24 h of cell adhesion, the culture medium was refreshed. The HK-2 cells were exposed to different concentration of H_2_O_2_ (0, 125, 200, 250, 500, 1000, 1500 μM) for 24 h after treatment with different concentration of FTZ (12.5, 25, 50, 100, 150, 200 μg/mL) for 1 h. Following the treatment, 10 μL CCK-8 (dark operation) was added to each well for 1 h at 37 °C. The absorbance was recorded at 450 nm with a microplate analyzer. The HK-2 cells after treatment with 200 μM of H_2_O_2_ and FTZ (0, 50, 100, 150 μg/mL) for 24 h were collected to further measure the levels of ROS, SOD, and LDH and the expression of related proteins with assay kits according to their manufacturer's instructions.

### Western blot assay

The kidney tissues or the collected HK-2 cells were homogenized in RIPA buffer (P0013B) containing a protease inhibitor cocktail. Total protein content was quantified by using a BCA Protein Assay Kit. Membranes were incubated with primary antibodies (Nrf2, HO-1, NQO1, Bax, Caspase3, Cleaved-caspase3, Bcl-2, GAPDH, and β-actin) overnight at 4 °C, followed by incubation with secondary antibodies for 1 h at room temperature. Then, the immune-reactive protein bands were visualized with clarity using western ECL substrate purchased from Bio-Rad Company. The protein band intensity was measured by using Bio-Rad image analysis (Bio-Rad, Hercules, CA, USA).

### TUNEL assay

The kidney tissues were fixed in 4% paraformaldehyde for 36 h, dehydrated with graded alcohol, embedded in paraffin, and then sliced into 4 μm-thick sections. The HK-2 cells cultured on the glass slice were also fixed with 4% paraformaldehyde. Cell apoptosis was detected using a TUNEL assay. The signal intensity of green fluorescence was observed under a fluorescence microscope.

### Statistical method

All statistical analyses were performed using GraphPad Prism 7.0. The results were recorded as the mean ± SEM, and the differences between the groups were analyzed by one-way ANOVA and t-test. *P* < 0.05 was considered to be a significant difference.

## Results

### FTZ alleviates the disorder of glucose-lipid metabolism

**Fig. 2:**
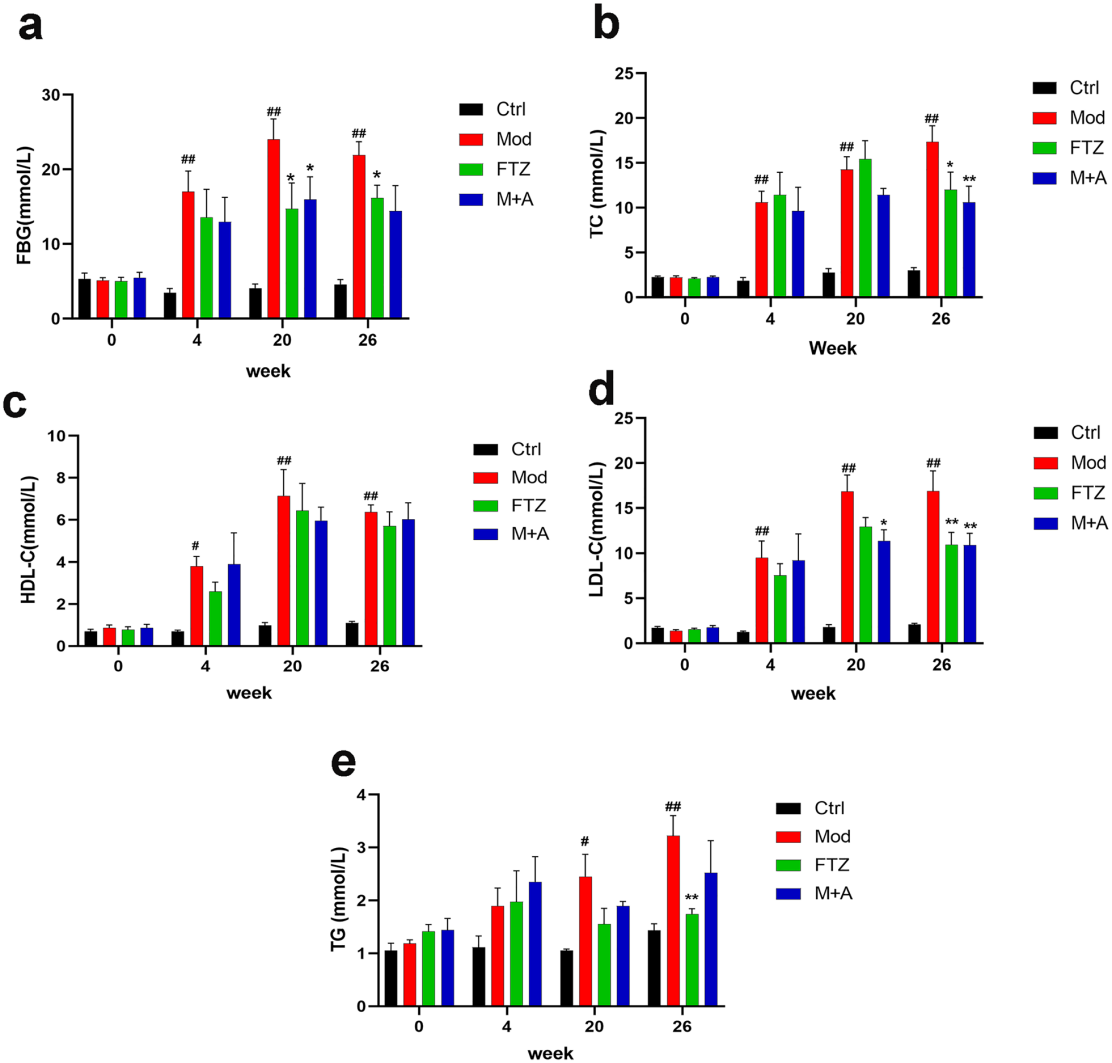
Effect of FTZ on glucose-lipid metabolism in DM-CHD minipigs. **a** Changes in serum levels of GLU. **b** Changes in serum levels of TC. **c** Changes in serum levels of HDL-C. **d** Changes in serum levels of LDL-C. **e** Changes in serum levels of TG. Values are the mean ± SEM. ^#^*P* < 0.05, ^##^*P* < 0.01(compared to Ctrl group); ^*^P < 0.05, ^**^P < 0.01 (compared to Mod group) (n = 4–5)

There was no difference between groups in the levels of GLU, TC, HDL-C, LDL-C, and TG of the minipigs at the beginning of the experiment. From 4 to 26 weeks, the levels of FBG, TC, HDL-C, LDL-C, and TG in the model group were significantly higher than in the control group. At 20 and 26 weeks, the levels of FBG, TC, LDL-C, and TG in the FTZ group were reduced significantly compared with the model group, while the HDL-C levels have no significant difference between the groups (Fig. 2). As a result, FTZ can alleviate the disorder of glucose-lipid metabolism.

### FTZ alleviates renal injury in DM-CHD minipigs

**Fig. 3:**
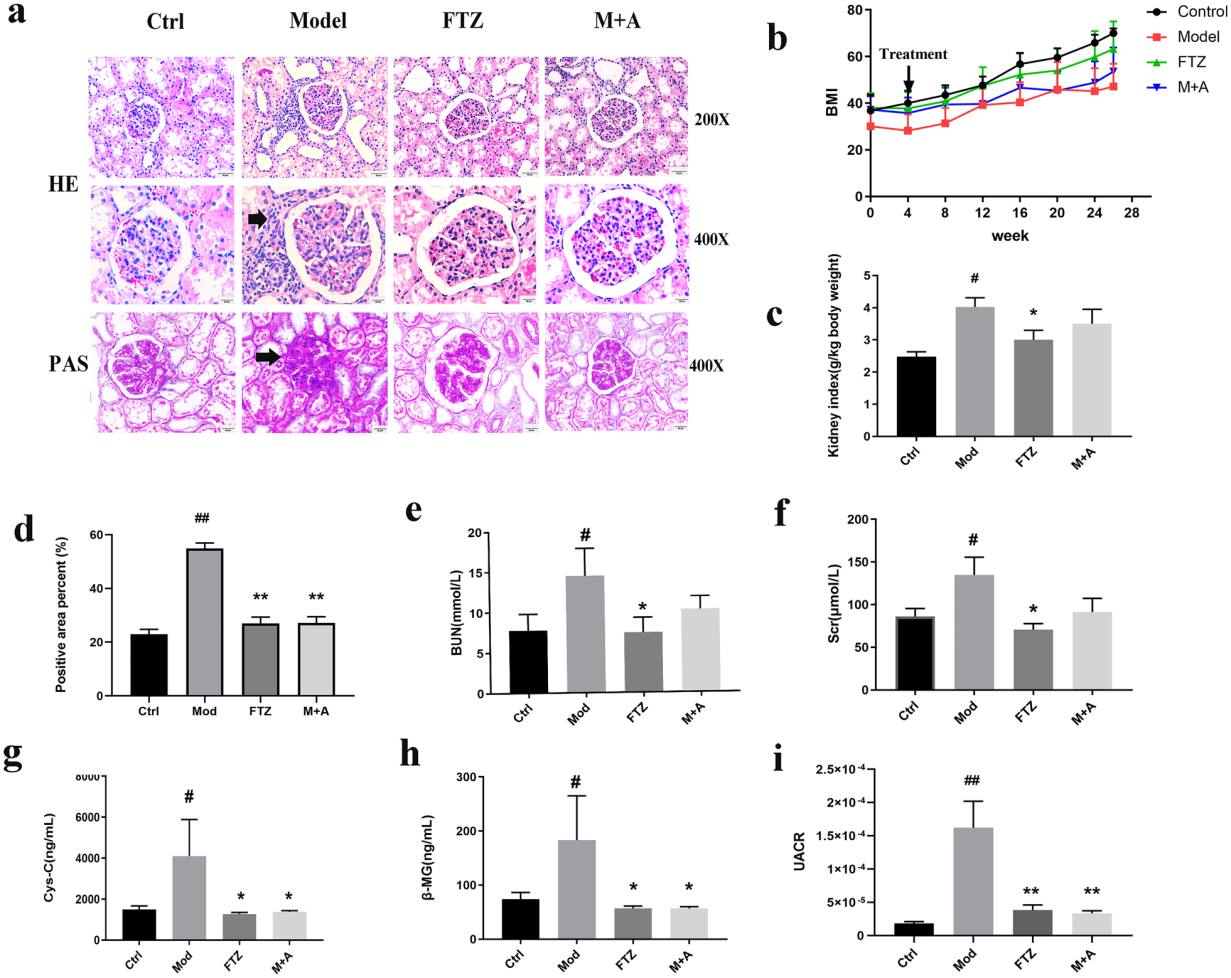
FTZ alleviates renal injury in DM-CHD minipigs. **a** The result of H&E staining (magnification × 200, × 400) and PAS staining (magnification × 400) in kidney tissue. **b** Changes in BMI. **c** Changes in kidney index. **d** The result of PAS staining analysis. **e**–**h** Changes in the serum levels of BUN, Scr, Cys-c, and β-MG. (i) Changes in the urine levels of UACR. Values are the mean ± SEM. ^#^*P* < 0.05, ^##^*P* < 0.01(compared to Ctrl group); ^*^P < 0.05, ^**^P < 0.01 (compared to Mod group). (n = 4–5)

We then investigated whether FTZ has a protective effect on renal injury in DM-CHD minipigs. The results of the H&E staining showed that in the model group, a large number of inflammatory cells infiltrated around the glomerulus, while no inflammatory cells were seen in the control group, and the number of inflammatory cells was decreased in the FTZ group. The results of the PAS staining demonstrated that the positive area in glomeruli and renal tubules increased significantly in the model group, indicating that there was a large amount of glycogen deposition, while it was decreased in the FTZ group and M + A group, which tended to the control group level (Fig. 3a, b). The BMI of the model group was lower than that of the control group and the kidney index of the model group was significantly higher than that of the control group. The BMI and kidney index of the FTZ group were improved and tended to be close to that of the control group (Fig. 3c, d).

The levels of BUN, Scr, Cys-C, β-MG, and UACR in plasma and urine were measured to evaluate renal function. The results showed that their levels of the model group were significantly higher than that of the control group, indicating the occurrence of renal injury. However, their levels were decreased in the FTZ group and M + A group, and the FTZ group showed better effect than the M + A group (Fig. 3e–i).

Therefore, it suggested that FTZ can alleviate renal injury in DM-CHD minipigs by improving renal function and suppressing the inflammatory and glycogen deposition pathological process.

### FTZ enhances the antioxidant capacity of the DM-CHD minipigs

**Fig. 4:**
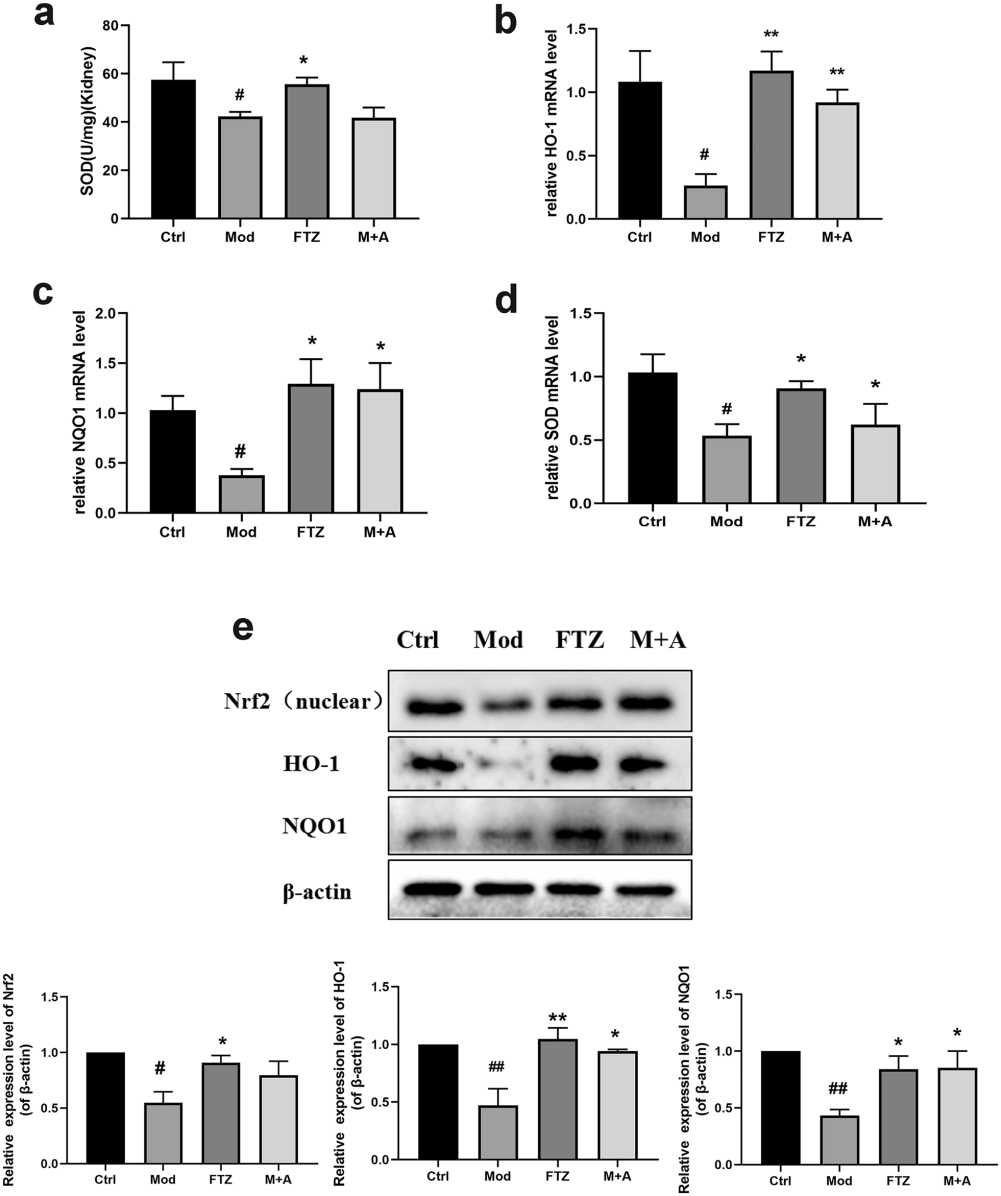
FTZ enhances the antioxidant capacity of the DM-CHD minipigs **a** The level of SOD in the kidney tissues. **b**–**d** The levels of HO-1, NQO1, and SOD mRNA in the kidney tissues. **e** The expression of Nrf2, HO-1, and NQO1 protein in the kidney tissues. Values are the mean ± SEM. ^#^*P* < 0.05, ^##^*P* < 0.01(compared to Ctrl group); ^*^P < 0.05, ^**^P < 0.01 (compared to Mod group) (n = 4–5)

The level of SOD in kidney tissues was detected to evaluate the effect of FTZ on antioxidant activity in the kidney. Therefore, we further investigated the effects of FTZ on SOD levels in kidney tissue of the DM-CHD minipigs. The kidney SOD levels in the model group were significantly decreased (*P* < *0.05*) compared with the control group. The kidney SOD levels in the FTZ group were significantly increased (*P* < *0.05*) compared with the model group (Fig. 4a). Meanwhile, the SOD mRNA level also has the same trend (Fig. 4d).

The protein and mRNA levels of HO-1, NQO1, and Nrf2 in the kidney tissue, which are oxidative stress regulatory factors, were detected by WB and PCR (Fig. 4b, c, e). The results showed that, in the model group, the mRNA expression of antioxidant HO-1, NQO1 and SOD was decreased, and the protein expressions of Nrf2, HO-1, and NQO1 were down-regulated in the kidney tissues, but in the FTZ group, the mRNA expressions of antioxidant HO-1, NQO1 and SOD were increased, and the protein expressions of Nrf2, HO-1 and NQO1 were also up-regulated in the kidney tissues. And M + A group showed an inferior effect to the FTZ group.

The above results suggested that FTZ held the potential to effectively enhance the antioxidant capacity to reduce the oxidative stress response in the kidney tissues of the DM-CHD minipigs.

### FTZ alleviates apoptosis of kidney tissue in DM-CHD minipigs

**Fig. 5:**
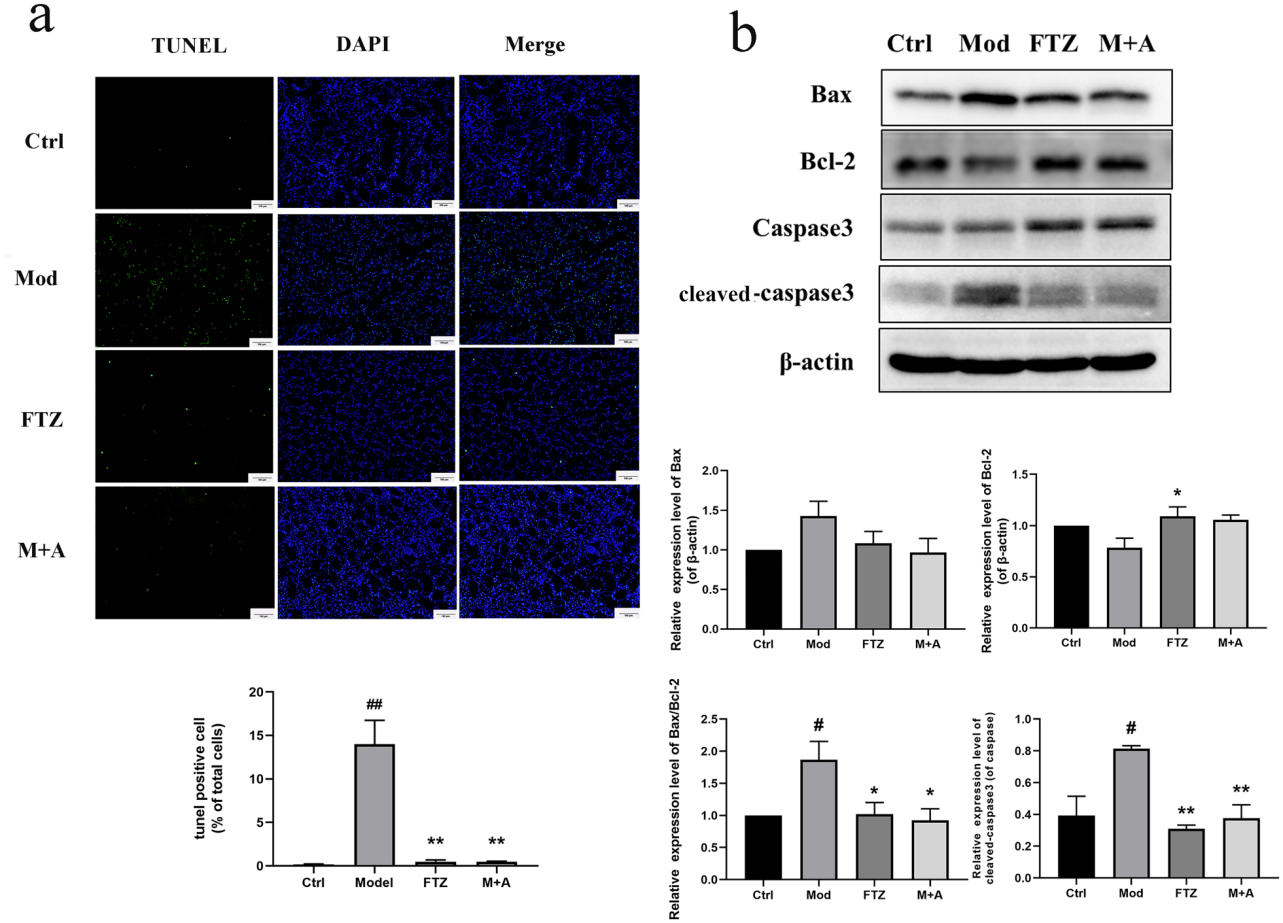
FTZ alleviates apoptosis of kidney tissue in DM-CHD minipigs. **a** TUNEL assay was performed on the kidney sections, and the green fluorescence pots are the apoptotic cells. **b** The expression of Bax, Bcl-2, Caspase 3, and Cleaved-caspase 3 protein in the kidney tissues. Values are the mean ± SEM. ^#^*P* < 0.05, ^##^*P* < 0.01(compared to Ctrl group); ^*^P < 0.05, ^**^P < 0.01 (compared to Mod group). (n = 4–5)

We further assessed the effects of FTZ on the apoptosis of the kidney of DM-CHD minipigs. As illustrated in Fig. 5a, a large number of obvious green fluorescence (*P* < *0.05*) was shown in the kidney tissues of the model group compared with that of the control group. In contrast, there was little green fluorescence in the FTZ group and M + A group (Fig. 5a).

Furthermore, we further assessed the effects of FTZ on the expression of the apoptosis-related protein in the kidneys, including the pro-apoptotic factor, Bax and Caspase 3, and anti-apoptotic factor, Bcl-2. The results showed that the expression levels of Bax/Bcl-2 and Caspase 3 were significantly increased, and Bcl-2 was significantly decreased in the kidneys of the model group (Fig. 5b). The expression levels of Bax/Bcl-2 and Caspase 3 were significantly decreased and the Bcl-2 was increased in the FTZ group. M + A group showed similar effects (both *P* < *0.05*).

These results demonstrated that the renal injury in DM-CHD minipigs was indeed ameliorated after FTZ treatment via down-regulating the pro-apoptotic protein Bax and Caspase 3, up-regulating the anti-apoptotic factor Bcl-2, respectively.

### ***FTZ ameliorates the cell oxidative injury induced by H***_***2***_***O***_***2***_*** in HK-2 cells***

**Fig. 6:**
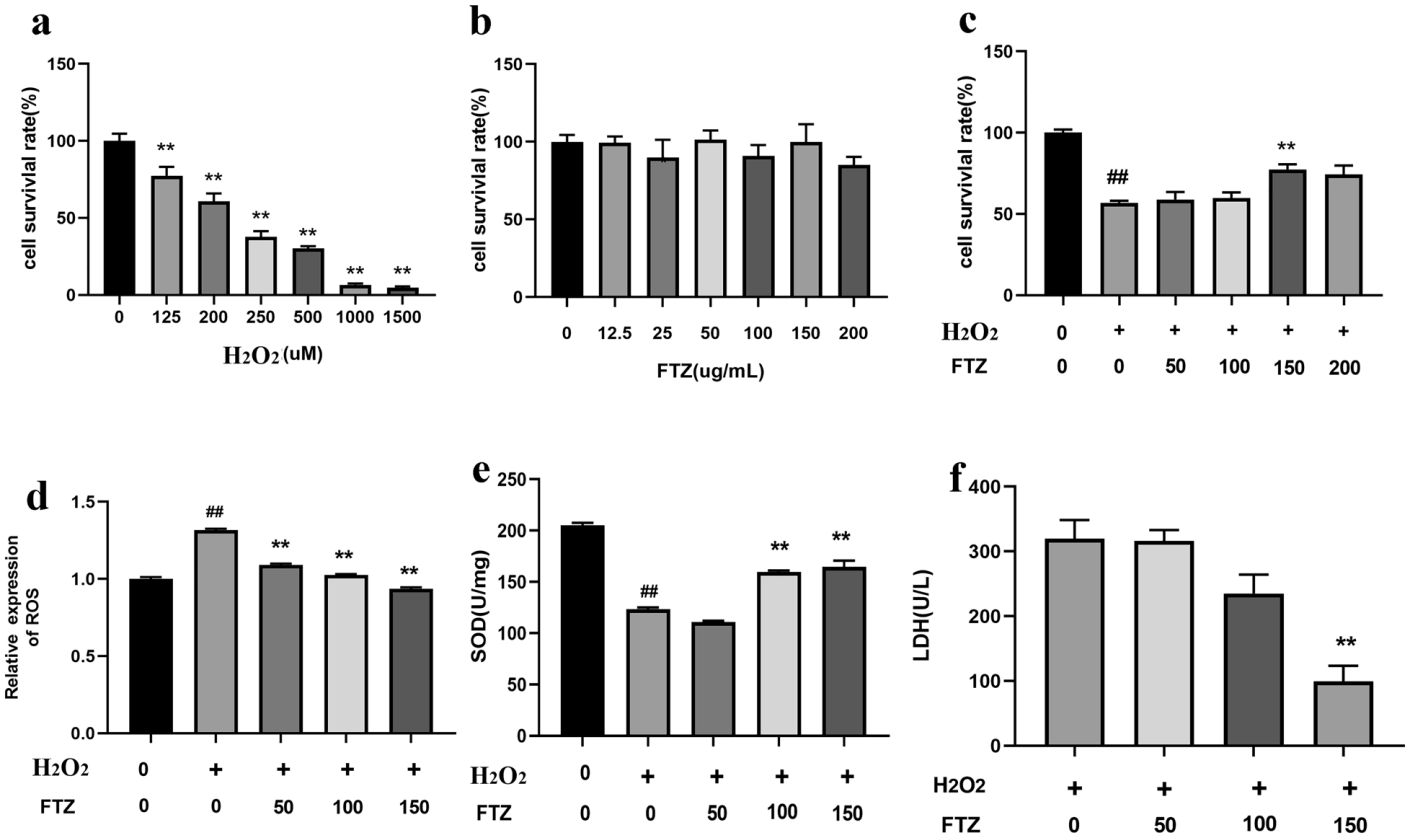
FTZ ameliorates the H_2_O_2_-induced oxidative injury in HK-2 cells. **a** Effects of H_2_O_2_ on HK-2 cell viability at different concentrations of H_2_O_2_. **b** Effects of different concentrations of FTZ on the viability of the HK-2 cells. **c** Effects of different concentrations of FTZ on the viability of HK-2 cells exposed to 200 µM H_2_O_2_. **d**–**f** Analysis of the levels of ROS, SOD, and LDH in the FTZ (0, 50, 100, 150 µg/mL) treated HK-2 cells exposed to 200 µM H_2_O_2_. Values are the mean ± SEM. ^#^*P* < 0.05, ^##^*P* < 0.01(compared to Ctrl group); ^*^P < 0.05, ^**^P < 0.01 (compared to Mod group) (n = 3)

To establish the oxidative injury model of HK-2 cells, different concentrations of H_2_O_2_ were used to induce HK-2 cells' oxidative injury. The cell viability assay with CCK-8 showed that with the concentration of H_2_O_2_ from 125 to 1500 μM, the viability of HK-2 cells was obvious decreased compared with the control group, and the viability of HK-2 cells was only about 50% when the concentration of H_2_O_2_ was 200 μM (Fig. 6a). Continuously increased concentration of FTZ in the range of 0–200 μg/mL did not cause a significant effect on the viability of HK-2 cells compared with the control group (Fig. 6b). To evaluate the effect of FTZ on oxidative stress, HK-2 cells were treated with 200 μM H_2_O_2_ and gradient concentration of FTZ at 50, 100, and 150 µg/mL.

FTZ could improve the decreasing cell viability induced by 200 μM H_2_O_2_ in a concentration-dependent way. When the concentration of FTZ was 150 μg/mL, the survival rate of HK-2 cells induced by 200 μM H_2_O_2_ reached the highest and there was a significant difference compared with the FTZ 0 μg/mL vehicle treatment (Fig. 6c). The above results showed that FTZ ameliorates the cell viability of H_2_O_2_-induced oxidative injury in HK-2 cells.

### FTZ increases oxidative resistance and suppressed ROS production in HK-2 cells

To investigate the protective effect of FTZ on oxidative injury in HK-2 cells, the contents of ROS, SOD, and LDH in the HK-2 cells were detected by kits. Results showed that 24-h H_2_O_2_ treatment significantly increased the contents of ROS and LDH, but decreased SOD content, while FTZ treatment on HK-2 cells significantly decreased ROS and LDH levels (Fig. 6d, f). Meanwhile, the expression of SOD was increased with the increase of FTZ concentration compared with the model group (Fig. 6e).

### FTZ protects HK-2 cells by activating Nrf2 pathway and inhibiting apoptosis

**Fig. 7:**
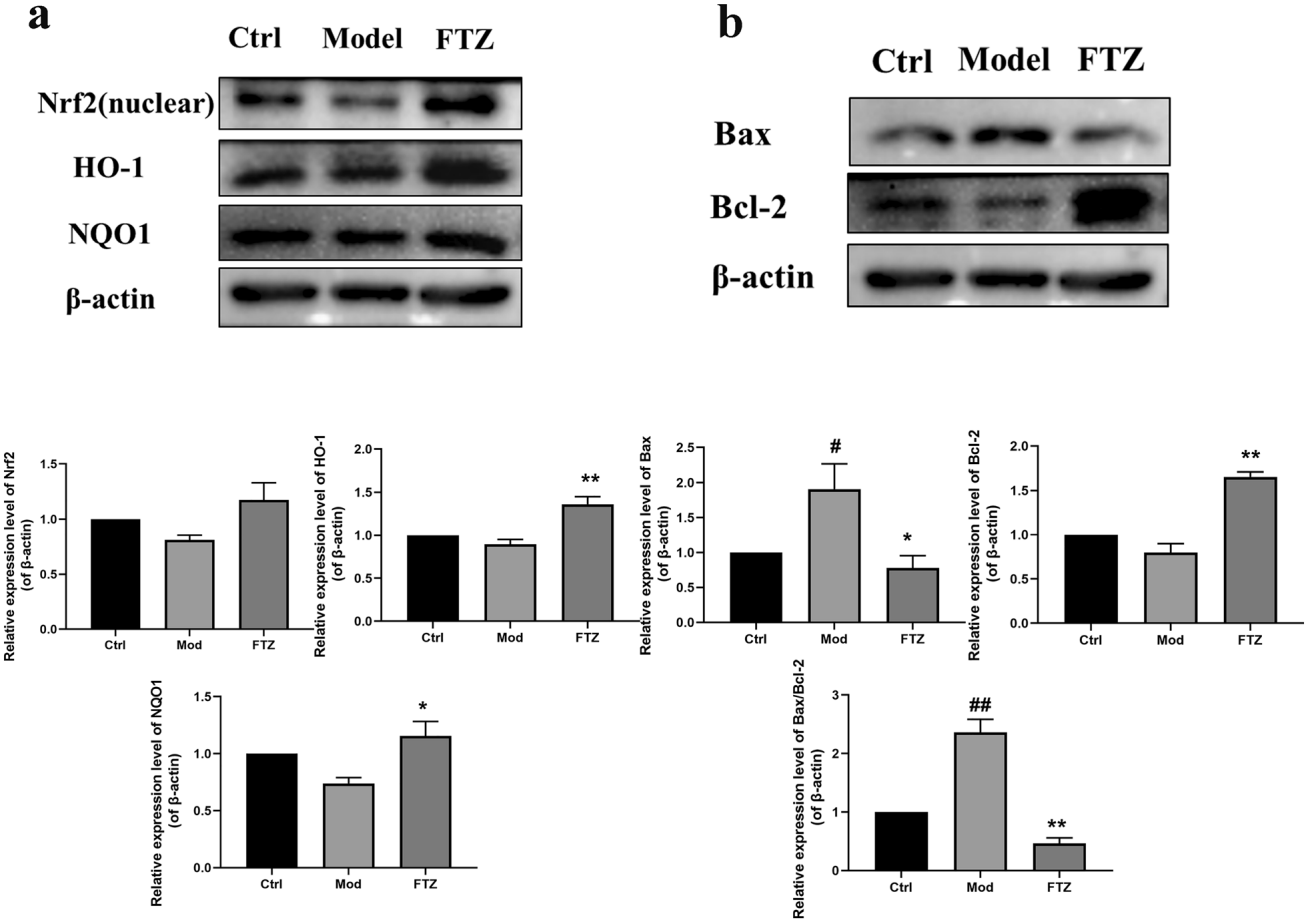
FTZ protects HK-2 cells by activating Nrf2 pathway and inhibiting apoptosis **a** The expression of Nrf2, HO-1, and NQO1 protein in the HK-2 cells. **b** The expression of Bax and Bcl-2 protein in the HK-2 cells. Model group: the HK-2 cells were exposed to 200 µM H_2_O_2;_ FTZ group: the HK-2 cells were exposed to 200 µM H_2_O_2_ for 24 h after 150 µg/mL FTZ treatment for 6 h. Values are the mean ± SEM. ^#^*P* < 0.05, ^##^*P* < 0.01(compared to Ctrl group); ^*^P < 0.05, ^**^P < 0.01 (compared to Mod group) (n = 3)

To explore the antioxidant mechanism of FTZ in HK-2 cells, the Nrf2, HO-1, and NQO1 in the HK-2 cells were further measured by western blot. As evidenced in Fig. 7a, the protein expression levels of Nrf2, HO-1, and NQO1 were notably decreased after H_2_O_2_ treatment compared with the control group. Not surprisingly, the FTZ reversed the decrease of Nrf2, HO-1, and NQO1 in the H_2_O_2_ treated HK-2 cells, indicating that FTZ effectively activates Nrf2 pathway to increase the expression of antioxidative factor HO-1 and NQO1.

We subsequently examined the expression of apoptosis-related proteins, Bax and Bcl-2, to investigate the potent anti-apoptosis mechanism. As shown in Fig. 7b, in the model group, the expression of Bax was increased and the expression of Bcl-2 was decreased, and the ratio of Bax/Bcl-2 was significantly increased compared with that of the control group (*P* < *0.01*). But FTZ decreased the expression of Bax and the ratio of Bax/Bcl-2(*P* < *0.01*), and increased the expression of Bcl-2.

These results indicate that FTZ has a protective effect on H_2_O_2_-induced HK-2 cell injury, and protects HK-2 cells by regulating Nrf2 to prevent oxidative stress and inhibiting the expression of apoptotic proteins, which further confirms the mechanism of action of FTZ on the kidney.

## Discussion

Nowadays, renal injury, a common microvascular complication of diabetes, known as DKD, has high morbidity and mortality, which seriously threatens human health [36, 37]. It has become one of the most important causes of end-stage renal disease (ESRD) [38, 39]. Therefore, the treatment of complications of renal injury is urgent. In a lot of scientific research, TCM has been widely used to treat and control diabetes and its complications such as renal injury [40]. The FTZ is an innovative prescription with multiple targets to comprehensively prevent and cure glucolipid metabolic disease [34, 36]. FTZ effectively inhibited coronary artery incrassation and protected the myocardium against injury in DM-CHD minipigs [35]. FTZ decreased proinflammatory cytokine levels and upregulated the protein expression of the PI3K/Akt pathway in the myocardium [35]. Therefore, based on our previous findings that FTZ has a protective effect on myocardial injury in DM-CHD minipigs, this paper aims to explore whether FTZ has a therapeutic effect on renal injury complicated with DM-CHD.

In diabetes, the renal tubules are affected by a metabolic disorder, inflammation, proteinuria, hemodynamic changes, and so on. The disorder presents with persistent albuminuria and a progressive decline in the glomerular filtration rate [39]. Reliable tests for diagnosis and monitoring include urine albuminuria and the estimated GFR (eGFR) [41]. In DM-CHD minipigs, there are a large number of inflammatory cells infiltration, glycogen deposition near glomerulus and apoptotic cells in the kidney tissue, and significantly increased levels of BUN, Scr, UACR, Cys-c, and β-MG. These results demonstrated that there is a renal injury in the DM-CHD minipigs. After FTZ treatment, the levels of BUN, Scr, UACR, Cys-c, and β-MG decreased, and the pathological process was suppressed. It is suggested that FTZ can alleviate renal injury in DM-CHD minipigs.

Over the past decade, many researchers have demonstrated that oxidative stress is intricately linked and a significant driver of these diabetic complications [42, 43]. So we detected the oxidative stress regulatory factors to explore the mechanism of FTZ prevention effect on renal injury. The results showed that the contents of SOD, HO-1 mRNA, NQO1 mRNA, and SOD mRNA in the kidney tissues were up-regulated in the FTZ group, and the protein expressions of Nrf2, HO-1, and NQO1 were significantly increased after FTZ administration. Apparently, FTZ could enhance the antioxidant capacity by activating Nrf2 pathway to relieve the oxidative stress in the kidney tissue.

Meanwhile, excessive production of ROS can lead to cell apoptosis, Caspase 3 is the prime executor of cell apoptosis and Bcl-2/Bax is the apoptosis regulator pair. Bcl-2 may block Caspase 3 to reduce cell apoptosis. Bax gene is the most important apoptotic gene. The Bax protein encoded can form a heterodimer with Bcl-2 and produce an inhibitory effect on Bcl-2 [44], so we further assessed the FTZ effect on cell apoptosis and its mechanism. We concluded that FTZ increased the expression of Bcl-2 protein and reduced the expressions of Bax and Caspase 3 protein, to alleviate apoptosis of the kidney tissue in DM-CHD minipigs. Subsequently, the role of FTZ in oxidative stress and apoptosis was verified in vitro with H_2_O_2_ induced HK-2 cellular injury. In this study, we disclosed that FTZ could elevate the expressions of Nrf2, HO-1, and NQO1 in the kidney tissues of DM-CHD minipigs and H_2_O_2_ induced HK-2 cells, which may directly contribute to its antioxidative effect to reduce cell apoptosis in the kidney tissues. Also, it may be owed to the enriching antioxidative compounds such as danshinone, protocatechuic acid, pinoresinol, salidroside, 5,7-dimethoxycoumarin and specnuezhenide, etc. in FTZ [29].

This study confirmed that excessive oxidative stress was one of the key pathological mechanisms of diabetic nephropathy. According to the theory of modern Chinese medicine, the product of oxidative stress is an important factor of "Zhuo" [48]. The FTZ created according to strategy "Tiao Gan Qi Shu Hua Zhuo" (modulating Gan, trigging key metabolic system to resolve pathogenic factors such as phlegm retention and dampness)" has the effects of regulating lipids, lowering blood sugar, improving endothelial cell function and anti-inflammatory [29, 30, 48]. Our study demonstrated that FTZ treatment could significantly improve glucose and lipid metabolism disorders, and renal function and reduce apoptosis. FTZ also increased the protein levels of SOD, Nrf2, HO-1, and NQO1, promoted antioxidant effects, down-regulated Bax, Caspase3, and up-regulated Bcl-2 to inhibit the apoptosis of kidney tissue and HK-2 cells. These results suggest that FTZ improves the pathology and function of diabetic nephropathy by rebalancing oxidative stress, and once again proves that regulating oxidative stress is one of the biological basis of FTZ's “Hua Zhuo”.

## Conclusion

In summary, our results demonstrated that FTZ can protect renal injury and improve glucolipid metabolism of DM-CHD minipigs, which are related to activating anti-oxidative stress to reduce apoptosis and inhibiting inflammation. It suggests that FTZ administration may be a promising therapeutic strategy for diabetic kidney disease.

## Data Availability

Please contact the author for data requests.

## Abbreviations

β- MG: β-Macroglobulin
BMI: Body Mass Index
BUN: Blood Urea Nitrogen
Cys-c: Cystatin
DKD: Diabetic Kidney Disease
DM: Diabetes Mellitus
DM-CHD: Diabetes Mellitus with Coronary Heart Disease
ESRD: End Stage Renal Disease
FTZ: Fufang-Zhenzhu-Tiaozhi
GFR: Glomerular Filtration Rate
GLU: Glucose
HDL-C: High Density Lipoprotein Cholesterol
HK-2: Human kidney-2
HO-1: Hypoeretin-1
Keap1: Kelch-like ECH-associated Protein 1
LDH: Lactate Dehydrogenase
LDL-C: Low Density Lipoprotein Cholesterol
MCP-1: Mono cytechemoattractant protein-1
NQO1: NAD(P)H Quinone Dehydrogenase 1
Nrf2: Nuclear Factor Erythroid 2–Related Factor 2
RAAS: Renin-angiotensin- aldosterone System
ROS: Reactive Oxygen Species
Scr: Serum Creatinine
SGLT2: Sodium-glucose cotransporter 2
SOD: Superoxide dismutase
STZ: Streptozotocin
TC: Total Cholesterol
TG: Triglyceride
T2DM: Type 2 Diabetic Mellitus
UACR: Urinary Albumin Creatinine Ratio
UALB: Urine Micro albumin
UCR: Urine creatinine
